# Rising Temperatures Advance Start and End of the Breeding Season of an Alpine Bird

**DOI:** 10.1002/ece3.70897

**Published:** 2025-02-02

**Authors:** Carole A. Niffenegger, Sabine M. Hille, Christian Schano, Fränzi Korner‐Nievergelt

**Affiliations:** ^1^ Swiss Ornithological Institute Sempach Switzerland; ^2^ BOKU University Vienna Austria

**Keywords:** alpine birds, breeding phenology, citizen science, climate change, high elevation, *Montifringilla nivalis*

## Abstract

Many bird species have advanced the start of the breeding season as a response to climate change. The duration of the breeding season and how it is affected by climate change are far less studied but are important for the re‐nesting potential. Re‐nesting includes both the replacement of a failed breeding attempt or breeding successfully multiple times within one season and can therefore impact fitness. Some species profit from an earlier start of breeding through a higher re‐nesting potential, whereas other species also advance the end of breeding season as conditions for breeding deteriorate. Here, we explored how temperature, precipitation, and snow conditions influence the start, end, and duration of the breeding season of a cold‐adapted high‐elevation songbird. We fitted generalized additive models with more than 12,000 citizen science observations of white‐winged snowfinches ( *Montifringilla nivalis* ) to estimate breeding phenology between 2006 and 2021. Our results indicate that higher prebreeding temperatures and reduced April precipitation were associated with an earlier start of breeding. However, later during the breeding season higher temperatures shortened the breeding season through an earlier end of the breeding season. Despite adjusting the timing of reproduction to prevailing environmental conditions, average temperatures during the breeding season increased over the 16‐year study period. Therefore, snowfinches need to move to higher elevations in order to track the thermal conditions. This study highlights the complex relationship between phenology and environmental conditions and illustrates how much the breeding conditions are currently changing for high‐elevation species.

## Graphical abstract

Snowfinches, a high‐elevation songbird, adjust the start and duration of breeding according to the prevailing environmental conditions. Despite these adjustments, average temperatures during the breeding season increased over the 16‐year study period. Therefore, snowfinches need to move to higher elevations in order to track the thermal conditions.

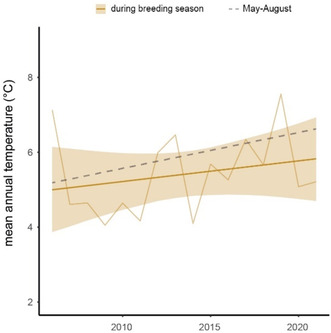

## 1 Introduction

Various environmental factors can influence the timing of reproduction. Rising spring temperatures are, for example, often associated with an earlier start of reproduction in birds (Dunn and Winkler 1999; Martin and Wiebe 2004; Pearce‐Higgins, Yalden, and Whittingham 2005; Madsen et al. 2007; Halupka, Dyrcz, and Borowiec 2008; Smith et al. 2024). Droughts, extensive snow cover, or heavy precipitation can instead delay the start of reproduction (Martin and Wiebe 2004; Madsen et al. 2007; Oppel et al. 2013; Smith et al. 2024). Some species do not only change when they start reproducing but also change the duration of the reproductive season. If conditions are still suitable after a successful breeding attempt or when a brood failed, some birds can re‐nest (Buckley, Andes, and Dabbert 2018; Halupka et al. 2021). The possibility of having more than one nesting attempt within a breeding season is described by the re‐nesting potential. The duration of suitable conditions can be altered by climate change. Ground foraging birds, for instance, tend to find less prey in desiccated soils (Resano‐Mayor et al. 2019). Therefore, we should consider the effect of environmental conditions on both the start and the duration of reproduction when we are assessing how climate change is impacting breeding phenology.

Changing temperature patterns are linked to reduced reproductive output in birds (Shipley et al. 2020; Taff and Shipley 2023). Such effects of changing environmental conditions are expected to be particularly pronounced in habitats with a short reproductive period and brief peaks in resource availability, such as at high elevation (Scridel et al. 2018). A short window of suitable conditions requires a tight link between the timing of reproduction and the environmental conditions to match resource peaks. High‐elevation habitats have a strong seasonality and high interannual variability in temperature and other environmental conditions such as precipitation and snow cover. Even during the reproductive season, sudden weather changes occur frequently at high elevation and include cold spells and summer snowfall. High‐elevation species thus require specific physiological and behavioral adaptations to cope with harsh conditions and a high flexibility to adjust to sudden changes in the environment (Martin and Wiebe 2004). Climate change affects high‐elevation habitats particularly strongly as the rate of warming is double the global average (Auer et al. 2007). The extent and speed of these changes might exceed the abilities of high‐elevation birds to adapt behaviorally or physiologically, and their special adaptation to cold environments may render them vulnerable to climate change (Martin and Wiebe 2004).

Here, we studied how environmental conditions influence the start, end, and duration of the breeding season of a cold‐adapted high‐elevation songbird species, the white‐winged snowfinch *Montifringilla nivalis* . Snowfinches are facultatively double brooded, and the breeding season duration varies considerably among populations and years (Grangé 2008; Strinella et al. 2011). Snowfinches are mostly resident with evidence of partial migration in harsh winters (Resano‐Mayor et al. 2020). During the nestling period, snowfinches mainly rely on invertebrate prey (Heiniger 1991) especially different types of invertebrate larvae that are abundant next to snowfield margins. Snowfields are therefore the preferred foraging ground of snowfinches while they tend to avoid high vegetation for foraging (Resano‐Mayor et al. 2019). Schano et al. (2021) showed that snowmelt advanced more strongly than the breeding phenology of snowfinches. As a consequence, the breeding phenology of snowfinches is lagging behind the earlier snowmelt. Our aim was to expand the knowledge about the impact of environmental conditions on the breeding season duration, as this is the next step to interpret potential consequences of changing phenology as response to climate change.

We used hierarchical generalized additive models (GAMs) to derive the breeding phenology from citizen science data. The data set consisted of more than 12,000 snowfinch observations from the Swiss Alps between 2006 and 2021. We used linear mixed effect models and sliding windows to explore the relationships between environmental conditions during the prebreeding and breeding period and the timing of reproduction. We expect that low temperatures or persistent snow cover act as environmental constraints and thus delay the start of breeding. At the same time, high temperatures may lead to rapid snowmelt and vegetation growth, which will decrease the availability of invertebrate prey. Lower food availability may shorten the time of suitable breeding conditions and may cause a shorter breeding season and a lower re‐nesting potential. Finally, we compared the rise in temperature during the breeding season of snowfinches in the study period with the general increase in temperature in the habitat to assess whether adjusting the timing of reproduction can mitigate the effects of climate change. Constant temperatures during the breeding season would suggest that snowfinches can track their thermal niche by adjusting the timing of reproduction to prevailing environmental conditions.

## 2 Materials and Methods

We used citizen science data from the Suisse Alps to calculate the start, end, and duration of the breeding season of snowfinches and subsequently explored the relationship between breeding phenology and temperature, precipitation, and snow conditions. Data were available from 2006 to 2021. For the analysis, we distinguished between four biogeographic regions of the Swiss Alps (northern Alps, eastern Alps, southern Alps, and western Alps) as these regions differ in their average environmental conditions (BAFU 2022).

### 2.1 Bird Data

We used bird observations from a validated citizen science database (ornitho.ch) in our analyses. The data set consisted of 12′441 observations of snowfinches in Switzerland that were reported between May 2006 and October 2021. During the breeding season (May to August) observers were encouraged to record behavioral information through codes (Appendix 1; Table A1), which indicates if a bird is breeding (e.g., if a bird is collecting food to provision nestlings). Higher breeding codes correspond to a higher probability of breeding. We used these breeding codes to divide the observations into two states: “likely breeding” vs. “nonbreeding.” We assigned the state “likely breeding” if the breeding code indicated courtship behavior (e.g., aerial displays, courtship feeding) or a higher breeding code. Observations of snowfinches occurring before May 1 and after August 31 were considered nonbreeding observations as there is no evidence for breeding activity outside this interval. This resulted in 1′869 observations with the state “likely breeding” and 10′572 observations in the state “nonbreeding.”

### 2.2 Environmental Data

#### 2.2.1 Average Temperature and Precipitation

We used daily average temperature and daily precipitation sum on a 100 m resolution. The data were interpolated from measurements of meteorological stations across Switzerland following Thornton, Running, and White (1997). The interpolated data were provided by the Swiss Federal Institute for Forest, Snow, and Landscape Research WSL (research unit land change science). We summarized the data per square kilometer and subsequently selected km^2^ with an average elevation between 1700 and 3300 m.a.s.l. corresponding to the elevational distribution of snowfinches in Switzerland (Knaus et al. 2018). Data were summarized per day and biogeographic region.

#### 2.2.2 Change of Mean Daily Temperature

We calculated the average daily temperature during the breeding season per year, that is, during the time, when the snowfinches were observed breeding in the specific year, and the yearly average temperature between May and August. We used daily average temperatures measured at a meteorological station in the eastern Alps (Weissfluhjoch, coordinates: 46°50′ N, 9°48′ E, elevation: 2691 m.a.s.l., source: MeteoSwiss) for this analysis.

#### 2.2.3 Snow Conditions

We used the daily snow cover fraction, that is, the percentage of snow cover per square kilometer. Data were obtained from Swiss snow monitoring stations and interpolated following Magnusson et al. (2014). These data were provided by the Institute for Snow and Avalanche Research SLF. We selected km squares in the Alps that had an average elevation between 1700 and 3300 m.a.s.l. and summarized the data per day and biogeographic region. For every region and year, we calculated the timing and duration of snowmelt. The timing of snowmelt was defined as the day when snow cover was at 50% coverage for the first time after snowmelt started. The duration of snowmelt was defined as the number of days between 95% and 5% of the maximum snow cover, as snow cover did not reach 100% in every year and in every region.

### 2.3 Statistical Analyses

All statistical analyses were carried out in the software R, version 4.2.2 (R Core Team 2020) and models were fitted in a Bayesian framework using the *brm* function from the package *brms* (Bürkner 2018). The convergence of the Markov chains was visually assessed using trace plots and with the Gelman‐Rubin Statistic (*Ȓ* < 1.01).

#### 2.3.1 Breeding Phenology Based on Generalized Additive Models

We used hierarchical generalized additive models (GAM) to derive phenology curves from citizen science data following Pedersen et al. (2019). The binomial state (“likely breeding” vs. “nonbreeding”) was the response variable. We fitted one model per year which included the day of year and the biogeographic region as predictors. We used cubic cyclic regression splines for the day of year to ensure that the fitted values for start and end (i.e., March and October) had a value of zero. For the biogeographic region, we used a group‐level smoother, so that the models fitted a global smoother which was shared among regions. Additionally, each region additionally had its own smoother, which allowed for different shapes of the phenology curve among regions. We used four chains with 8′000 iterations each and a thinning of four. Start day, end day, and breeding season duration were extracted from the posterior distribution of the expected proportion of likely breeding observations. Start of the breeding season was defined as the day of the year when the expected proportion of likely breeding observations exceeded 5% for the first time, and the end of breeding was defined as the day when it dropped below 5%. The breeding season duration was calculated as the number of days between the start and end of the day. We did not account for spatial or temporal autocorrelation in the GAMs as visual assessments of the semi‐variograms and acf‐plots indicated no patterns of autocorrelation in the residuals.

**FIGURE A1:**
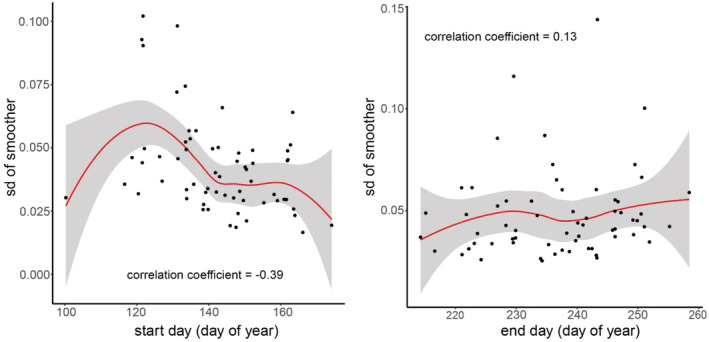
Uncertainty of smoother plotted against the calculated start day (left) and end day (right) of the breeding season. The absence of a clear pattern and the Pearson correlation coefficients indicate that there is no systematic bias of smoother uncertainty on the calculated start and end date of reproduction.

We conducted a sensitivity analysis to test how much the estimated duration of breeding season depends on the threshold to define start and end date by using alternative thresholds between 1% and 12%. The duration of breeding season did change in absolute value depending on the threshold, but the results were highly correlated (Pearson correlation coefficient above 0.89). The high Pearson correlation coefficients indicate that the selected thresholds gave robust indicators for the start, end, and duration of the breeding season. We assessed if uncertainty of the smoother would influence the calculated start and end date of the breeding season visually and by calculating the Pearson correlation coefficients. The results indicate that the uncertainty of the smoother did not bias the calculated start and end date of reproduction (Appendix 2; Figure A1).

#### 2.3.2 Identifying Critical Time Windows for Temperature and Precipitation

We used a sliding window approach to identify the time windows when temperature and precipitation correlate most strongly with start and end of the breeding season. We used a minimum window length of 7 days and fitted windows between April 1 and June 30 for the start of the breeding season and windows between April 15 and August 15 for the end of the breeding season. We selected these time windows based on the earliest brood of snowfinches that was recorded (own monitoring data). This brood hatched on April 30, and we expect that environmental cues that influence the timing of breeding would be most relevant in the weeks before. Similarly, we assumed that cues influencing re‐nesting would be most relevant after finishing the first breeding cycle. To reduce computing time, we slid windows by 2 days. For every time window, we calculated the Pearson correlation coefficient between the start or end of the breeding season and the mean temperature or precipitation days (i.e., the number of days within a window with at least 1 mm of precipitation). When calculating many correlations, some correlations will be high just by chance due to random scatter in the data (van de Pol et al. 2016). Therefore, we calculated the standard error of each correlation and only considered correlation coefficients with high certainty which are naturally also the correlations with the highest absolute values.

#### 2.3.3 Start, End, and Duration of the Breeding Season

We used linear mixed effect models to explore the effect of environmental conditions on breeding phenology. We picked the time window from the sliding window analysis with the highest absolute value as a predictor for the linear model. We fitted three separate models, which had either the start, end, or duration of the breeding season as a response variable. We used the temperature and precipitation during critical windows (i.e., time windows with the strongest positive and negative correlation based on sliding windows) and timing and duration of snowmelt as fixed effects. We included an interaction between temperature and precipitation as we expected the combined effect to be stronger on timing of reproduction, that is, more than additive as heavy precipitation in combination with low temperatures might be particularly energy demanding for the birds. To reduce model complexity, we only kept the interaction in models, where we could visually see an interactive effect in a partial effects plot. Year and biogeographic regions were included as random effects in all models (model structure: Appendix 3; Table A2). We used four Markov chains and 2′000 iterations to fit these models.

#### 2.3.4 Change of Mean Daily Temperature Across Years

We used linear models with a Gaussian error distribution to assess how the mean daily temperature changed during the breeding season and during summer, respectively. Mean daily temperature per year was the response variable and year was the explanatory variable. We used four Markov chains and 2′000 iterations to fit this model.

## 3 Results

The duration of the breeding season of snowfinches and the environmental conditions varied considerably between years and regions. Our results indicate that the average duration of breeding during the study period was 94 days and varied between 51 days (2019 in the southern Alps) and 132 days (2015 in the western Alps). The timing of snowmelt varied by up to 2 months between the earliest and latest year and the snowmelt lasted between 23 and 77 days.

### 3.1 Identification of Relevant Time Periods

**FIGURE 1:**
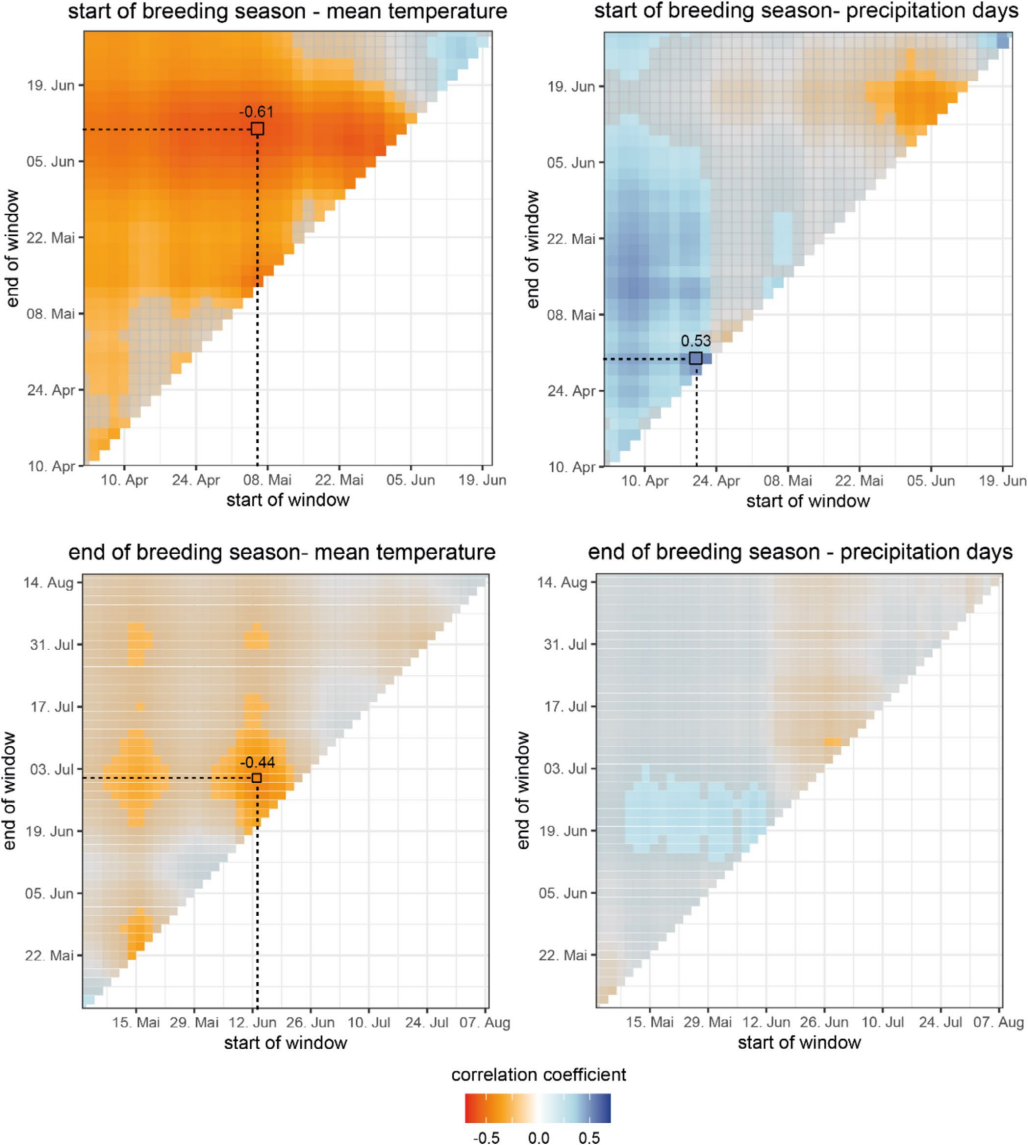
Results from the sliding window analysis. Every pixel represents one time window of which the start and the end is indicated on the x‐ and y‐axes. The colors reflect the value of the correlation coefficient between the start/end of the breeding season and the environmental variable for each time window. We used windows of a minimum length of 7 days and calculated the Pearson correlation coefficients of the start of breeding and end of breeding, respectively, with the mean temperature and the number of precipitation days within a specific window. Gray‐shaded areas indicate a high uncertainty of the correlation coefficients with uncertainty intervals overlapping zero. The black rectangles indicate the windows with the highest absolute correlation coefficients that were used in the linear regression about the influence of temperature, precipitation, and snow on the start and end of the breeding season. Numbers indicate the Pearson correlation coefficient of the window with the highest absolute value in each panel.

The sliding window approach identified three relevant time periods: two for temperature and one for the number of precipitation days. The daily average temperature between May 5 and June 10 was identified to be most strongly correlated with breeding start (Figure 1), whereas the average temperature between June 14 and June 30 was most strongly associated with the end of breeding (Figure 1). Furthermore, the number of precipitation days between April 19 and April 29 was negatively correlated with the start of breeding, that is, more days with precipitation delayed the start of reproduction of snowfinches (Table 1). Precipitation had no clear effect on the end of the breeding season (Figure 1) indicated by correlation coefficients close to zero.

**TABLE 1:** Parameter estimates of the linear mixed effect model for the start of the breeding season of snowfinches ( *Montifringilla nivalis* ).

	Standardized coefficients	Lower 95% CI	Upper 95% CI	Unstandardized coefficients	Lower 95% CI	Upper 95% CI
Fixed effects						
Intercept	142.3	132.9	151.7			
Temperature (°C) (May 5–June 10)	−9.60	−14.8	−3.17	−1.61	−10.87	7.49
Precipitation days (April 19–April 29)	4.13	−0.08	8.05	3.71	−0.14	7.53
Snowmelt timing (day)	−0.52	−4.88	4.25	−0.04	−0.37	0.30
Temperature: precipitation	−1.83	−4.79	1.26	−0.61	−1.62	0.45
Random effects						
Biogeographic regions (SD)	8.46	2.66	20.03	8.62	2.33	20.83
Year (SD)	6.30	2.12	11.94	6.24	2.05	11.72

*Note:* Standardized coefficients to compare effect sizes of variables; unstandardized coefficients to assess the effect on the response per unit change of the explanatory variable. Estimates of random effects indicate the extent of variation that is explained by biogeographic region and year, respectively.

### 3.2 Start of the Breeding Season

**FIGURE 2:**
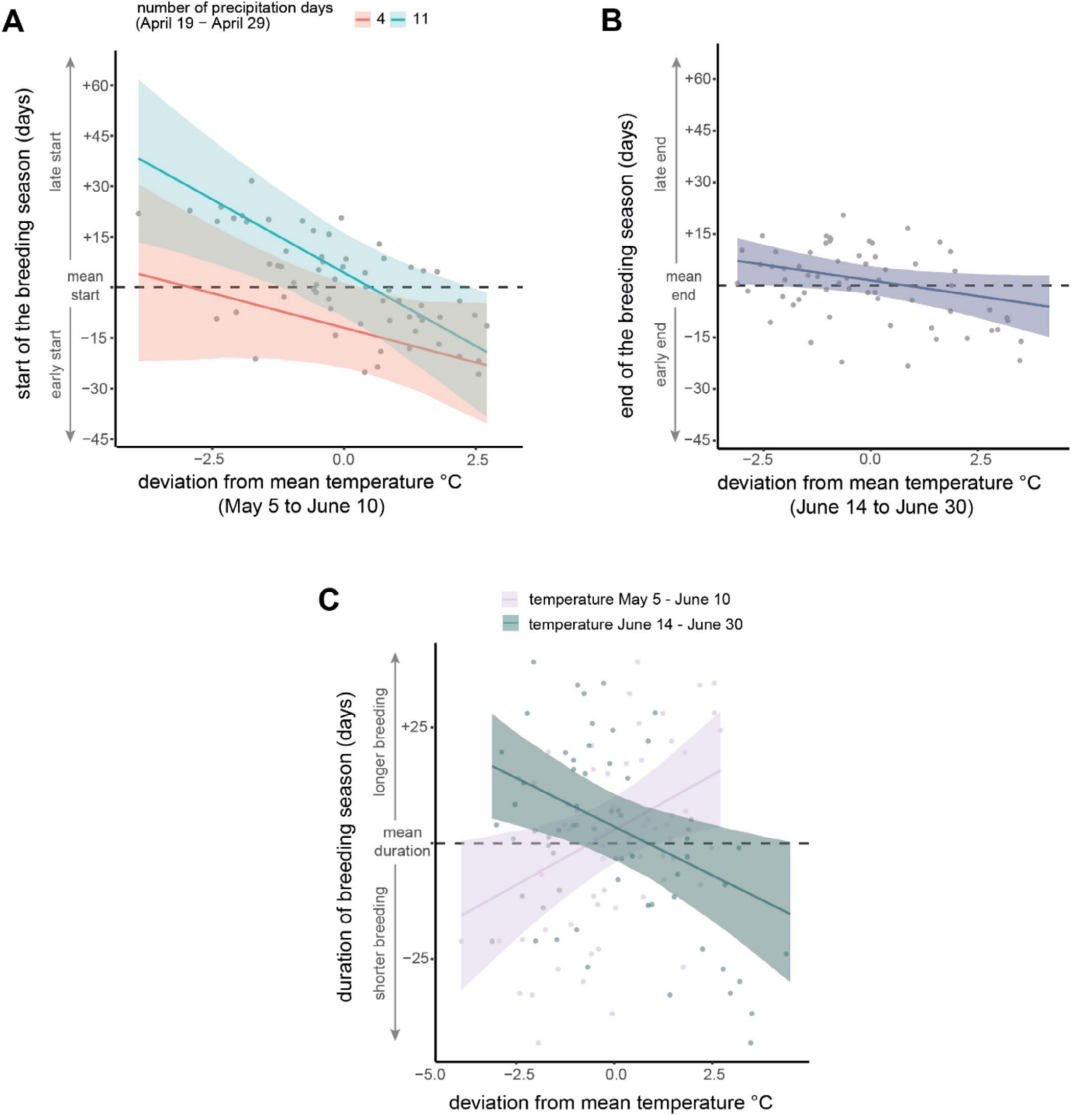
(A) Relationship between mean daily temperature during the critical time window from May 5 to June 10 and the breeding start of snowfinches ( *Montifringilla nivalis* ). Change in breeding start is expressed as deviation from the mean start (dashed line) with positive values corresponding to a delayed start and negative values to an earlier start of reproduction. The red line shows the relationship between temperature and the start of breeding in dry conditions with 4 days of precipitation between April 19 and April 29 (minimum number of precipitation days during the critical time window). The blue regression line shows the relationship in wet conditions with 12 precipitation days (maximum number of precipitation days during the critical time window). (B) Relationship between mean daily temperature during the critical time window from June 14 to June 30 and the end of the breeding season as deviation from the mean end date (dashed line). (C) Relationship between the mean daily temperature during the two critical time windows (purple: prebreeding; breeding: during breeding) and the breeding season duration as deviation from the mean breeding season duration (dashed line). The shaded area around the regression line depicts the 95% credible interval.

Our results show that higher prebreeding temperature was correlated with an earlier breeding start (Figure 2, Table 1). The effect of temperature was enhanced by precipitation in April. A combination of low temperature and many precipitation days was associated with a delay of breeding start by up to 39.0 days (95% CrI: 14.0–63.3 days). In contrast, at high temperatures, the effect of precipitation was small (Figure 2). For average precipitation conditions, the start of the breeding season advances by 6.0 days/+1°C (95% CrI: 1.8–9.4 days/+1°C). The effect of the snowmelt timing was small in comparison to the effect of temperature and precipitation (Table 1). If snowmelt was 10 days earlier, the start of breeding was 0.41 days delayed (95% CrI: 3.0 to −3.7 days).

### 3.3 End of the Breeding Season

Higher temperatures during the breeding season were associated with an earlier end of breeding (Figure 2B, Table 2). For every 1°C higher average temperature, the breeding season ended 1.8 days earlier (95% CrI: 0.2–3.6 days), given the timing and duration of snowmelt were constant. The effects of snowmelt timing and snowmelt duration were small in comparison to the temperature effect (Table 2). For every 10 days earlier snowmelt, breeding season ended 0.7 days earlier (95% CrI: −1.8 to 3.1 days), and a 10 days shorter snowmelt period was associated with a 2.4 days earlier end of breeding (95% CrI: 0.15–4.7 days).

**TABLE 2:** Parameter estimates of the linear mixed effect model for the end of the breeding season of snowfinches ( *Montifringilla nivalis* ).

	Standardized coefficients	Lower‐95% CI	Upper‐95% CI	Unstandardized coefficients	Lower‐95% CI	Upper‐95% CI
Fixed effects						
Intercept	236.6	232.4	240.8			
Temperature (°C) (June 14–June 30)	−3.65	−6.83	−0.20	−1.82	−3.57	−0.02
Snowmelt timing (day)	0.99	−2.38	4.09	0.08	−0.18	0.31
Snowmelt duration	2.52	−0.21	5.29	0.24	−0.00	0.47
Random effects						
Biogeographic region (SD)	2.49	0.08	8.93	2.42	0.09	8.84
Year (SD)	4.02	0.36	8.45	4.23	0.50	8.65

*Note:* Standardized coefficients to compare effect sizes of variables; unstandardized coefficients to assess the effect on the response per unit change of the explanatory variable. Estimates of random effects indicate the extent of variation that is explained by biogeographic region and year, respectively.

### 3.4 Breeding Season Duration

Our results suggest that temperature and precipitation during the prebreeding period and temperature during the breeding season were associated with changes in the breeding season duration (Figure 2C, Table 3). For every 1°C temperature increase between May 5 and June 10, the breeding season was 4.8 days longer (95% CrI: 1.0–8.3 days/+1°C; Figure 2C). Precipitation days in April shortened the breeding season by 2.4 days for every additional day with precipitation between April 19 and April 29 (95% CrI: 0.1–4.8 days). In contrast, higher temperatures during the breeding season (June 14–June 30) shortened the breeding season by 4.2 days/+1°C (95% CrI: 1.2–7.0 days/+1°C).

**TABLE 3:** Parameter estimates of the linear mixed effect model for the breeding season duration of snowfinches ( *Montifringilla nivalis* ).

	Standardized coefficients	Lower 95% CI	Upper 95% CI	Unstandardized coefficients	Lower 95% CI	Upper 95% CI
Fixed effects						
Intercept	93.81	87.26	100.6			
Temperature (°C) (May 5–June 10)	7.07	1.42	12.71	5.35	−5.77	16.47
Precipitation days (April 19–April 29)	−5.01	−9.74	0.02	−2.24	−6.77	2.22
Temperature (°C) (June 14–June 30)	−8.24	−13.50	−2.48	−4.20	−7.04	−1.19
Temperature (May 5–June 10): Precipitation days (April 19–April 29)	−0.23	−4.15	3.73	−0.08	−1.40	1.24
Random effects						
Biogeographic region (SD)	2.96	0.08	11.87	3.31	0.06	13.85
Year (SD)	10.46	5.89	17.26	10.49	5.87	17.15

*Note:* Standardized coefficients to compare effect sizes of variables; unstandardized coefficients to assess the effect on the response per unit change of the explanatory variable. Estimates of random effects indicate the extent of variation that is explained by biogeographic region and year, respectively.

### 3.5 Change in Mean Temperature Across Years

**FIGURE 3:**
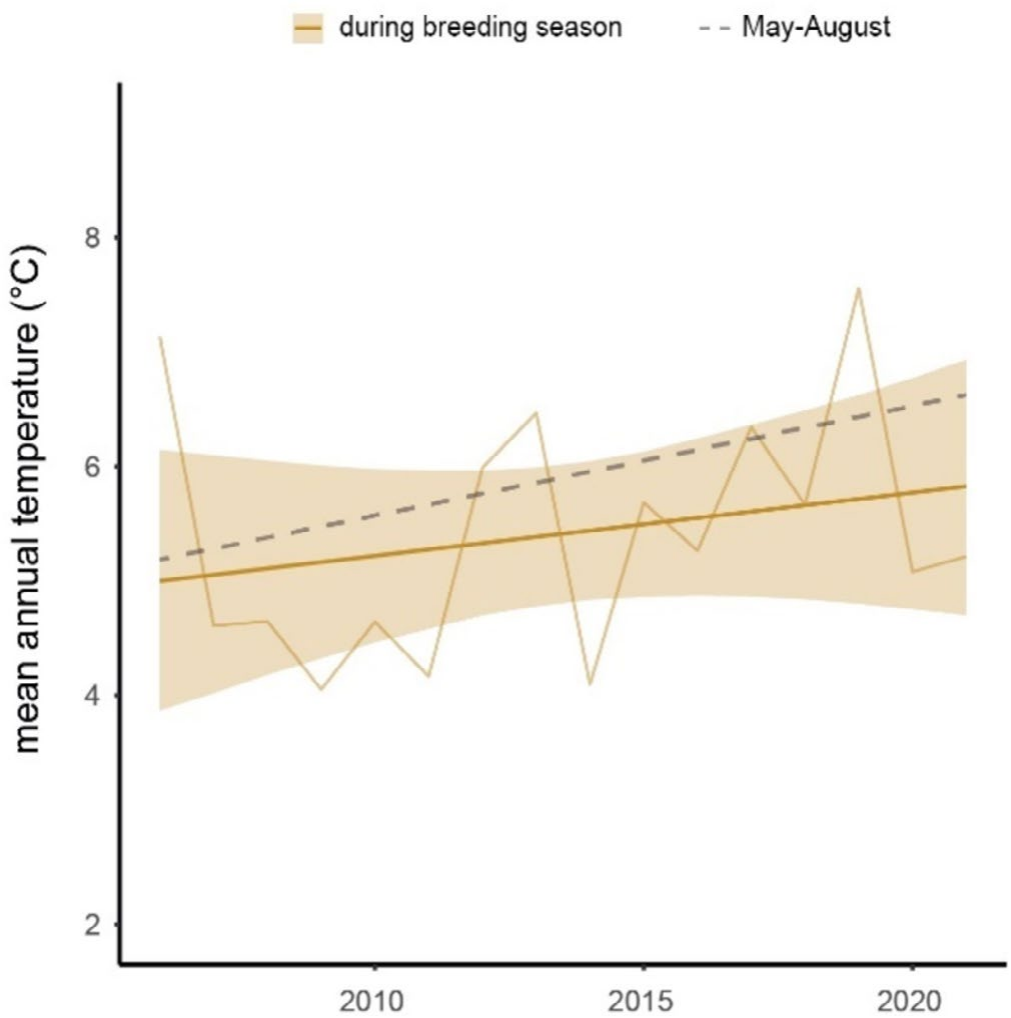
Changes in mean daily temperature during the study period from 2006 to 2021. Yellow regression line displays the change in mean daily temperature during the breeding season, that is, the change in conditions birds experienced despite adjusting the timing of reproduction. The gray dashed line indicates the mean temperature change of summer temperature (May–Augst). Results are based on the meteorological station “Weissfluhjoch” and the timing of reproduction in the eastern Alps. Shaded areas display the 95% credible intervals.

The mean daily temperature during the breeding season of snowfinches increased by 0.8°C during the study period (95% CrI: −1.2°C to 2.7°C), while the mean temperature in summer (May—August) increased by 1.5°C (CrI −0.01°C to 3.0°C). However, the between‐year variability average temperature was high (Figure 3).

## 4 Discussion

Climate change is altering the environmental conditions during the breeding season which changes the timing of reproduction in many bird species. Here, we showed how temperature, precipitation, and snow conditions influence the breeding phenology of a high‐elevation songbird. Our results indicate that high prebreeding temperatures led to an earlier start of breeding in snowfinches while high temperatures during the breeding season were associated with an earlier end of breeding and therefore shorter breeding season. We showed that the average temperature during the breeding season increased by 0.8°C during the 16‐year study period but this increase was less pronounced than the increase in summer temperature at the same location. In contrast to our expectation, we did not find a clear effect of snowmelt timing or snowmelt duration on the breeding phenology of snowfinches.

The differential effect of temperature on the start and end of the breeding season suggests that temperature is affecting the breeding phenology through several mechanisms. High prebreeding temperatures may alleviate environmental constraints, whereas above‐average temperatures during the breeding season might deteriorate breeding conditions. In birds, an earlier start of breeding with higher temperatures has been reported frequently (e.g., Townsend et al. 2013; Lv et al. 2019; Shipley et al. 2020; Kimmitt et al. 2022). The few existing studies on the effects of environmental conditions on the end of the breeding season also showed an advanced end of breeding with higher temperatures (Tarwater and Arcese 2018; Lv et al. 2019).

During the prebreeding season, the habitat of snowfinches is typically mostly snow‐covered (Schano et al. 2021; Müller et al. 2023; Niffenegger et al. 2023), and Schano et al. (2021) showed that low spring temperature delayed the hatching date of snowfinches. Low temperatures during this period are expected to delay snowmelt and thus lead to late vegetation green‐up and lower availability of invertebrate prey (Bolduc et al. 2013; Resano‐Mayor et al. 2019). Delayed access to nesting material and prey might prevent birds from brood initiation. Our results indicate that precipitation days at the end of April further reinforced the temperature effect with the latest start in years with low temperatures and frequent precipitation. Similarly, Schano et al. (2021) showed that heavy precipitation in April and May delays the hatching date of snowfinches. The combination of precipitation and low temperature poses energetic costs through reduced resource availability and increased cost of self‐maintenance (Grabowski et al. 2013; Gardner et al. 2018; Taff and Shipley 2023). Such conditions can thus negatively affect the body conditions and energy stores available for breeding and therewith delay the breeding start (Buckley, Andes, and Dabbert 2018) or even lead individuals to skip breeding entirely (Heiniger 1991).

Early end of breeding with higher temperatures during the breeding season may indicate deteriorating breeding conditions which reduce the renesting potential and shorten the breeding season. High temperatures lead to faster vegetation development (Frei, Ghazoul, and Pluess 2014). As a consequence, vegetation is dense and high shortly after snowmelt and prey availability for ground foraging birds is thus reduced (Resano‐Mayor et al. 2019; Barras et al. 2020; Müller et al. 2023). Moreover, high temperatures can cause heat stress for parents and offspring. Cold‐adapted species typically have low evaporative cooling capacity (O'Connor et al. 2021), which renders them vulnerable to heat stress even at relatively low temperatures. Negative impacts can arise if, for instance, provisioning rates are reduced or if nest cavities overheat. However, so far, very little research has been conducted on the effect of high temperature events on high elevation species even though the number of extreme temperature events is predicted to increase as a consequence of climate change.

In birds, the duration of the breeding season has been shown to correlate with reproductive success (Nagy and Holmes 2005; Tarwater and Arcese 2018; Mingozzi et al. 2022). The opposite is true if unfavorable environmental conditions impede the start of reproduction. Snow buntings ( *Plectrophenax nivalis* ), for instance, delay the start of breeding in years with late snowmelt, which reduced the renesting potential (Watson 1994). Similarly, we expect that snowfinches have a lower renesting potential in years with a short breeding season. However, more data on renesting in snowfinches and its fitness consequences are needed to get a more detailed understanding of how phenological changes are linked to the population dynamics of this high‐elevation specialist.

In contrast to our expectations, we did not find clear effects of the timing and duration of snowmelt on the breeding phenology of snowfinches. Schano et al. (2021) found that snowmelt advanced overall during the last two decades but especially at low elevation, snowfinch hatching dates did not follow the advancing snowmelt. Other studies from snow‐dominated habitats instead showed a link between snow conditions and breeding phenology (Madsen et al. 2007; Wilson and Martin 2010). Many species rely on snow‐free patches for foraging and profit from low sward height and open ground directly after snowmelt. These conditions provide optimal foraging opportunities for ground‐foraging birds (Resano‐Mayor et al. 2019; Barras et al. 2020; Müller et al. 2023). In our study, we summarized the snow conditions on the scale of biogeographic regions while most studies that found an effect of snow conditions operated on much smaller scales. Some studies specifically found that birds choose foraging or nesting microhabitat according to snow conditions (Brambilla et al. 2017; Schano et al. 2021; Müller et al. 2023; Niffenegger et al. 2023) which can vary substantially across small spatial scales due to topography and patterns of snow accumulation. The average snow condition over a large scale as used in this study might therefore not accurately represent the snow conditions at breeding sites, which may have led to the lack of association between snow conditions and breeding phenology in our data. In contrast, spatial correlation of temperature is large, and therefore, the variance of average temperature over large scale might represent local variance in temperature good enough to predict the timing of reproduction. Alternatively, snow conditions might influence primarily the breeding success, that is, the number of offsprings rather than the phenology.

Overall, our results indicate that snowfinches have a flexible timing of reproduction and adjust their phenology to environmental conditions. Start of breeding and the breeding season duration varied considerably during the 16‐year study period, and the timing of reproduction was strongly linked to temperature. Snowfinches were able to partially buffer the increase in summer temperature during the study period as indicated by less pronounced temperature increase during the snowfinch breeding season compared to the overall increase in summer temperature. However, despite adjusting the start and end of reproduction to temperature, they still experienced an increase of 0.8°C if they kept breeding at the same elevation. To track the temperature niche during breeding, snowfinches therefore would need to move to higher elevation which is also observed (Knaus et al. 2018). Population declines were most pronounced at the lower parts of the elevational distribution and breeding records below 1800 m of elevation became rare in recent years (Knaus et al. 2018).

Our results illustrate the complex relationship between environmental conditions and the breeding phenology of a high‐elevation specialist. The results highlight the potential sensitivity of snowfinches to climate change and showcase how citizen science data can be used to infer phenology in the absence of structured long‐term monitoring programs.

## Author Contributions

**Carole A. Niffenegger:** conceptualization (equal), data curation (lead), formal analysis (lead), writing – original draft (lead), writing – review and editing (lead). **Sabine M. Hille:** conceptualization (equal), supervision (equal), writing – review and editing (supporting). **Christian Schano:** conceptualization (supporting), data curation (supporting), formal analysis (supporting), writing – review and editing (supporting). **Fränzi Korner‐Nievergelt:** conceptualization (equal), formal analysis (supporting), project administration (lead), supervision (equal), writing – original draft (supporting), writing – review and editing (supporting).

## Conflicts of Interest

The authors declare no conflicts of interest.

## Data Availability

R codes and data are available on vogelwarte.ch Open Repository and Archive (https://zenodo.org/records/14445224).

## Appendix 1

**TABLE A1:** Behavioral codes adapted from https://www.ornitho.ch/index.php?m_id=41&langu=en.

**Possible breeding**		
1	Species heard or observed within potential breeding season, but outside of suitable breeding habitat	“nonbreeding”
2	Species heard or observed within potential breeding season and in suitable breeding habitat	“nonbreeding”
3	Territorial behavior such as singing or aggressive interaction indicating that a territory is defended	“nonbreeding”
**Probable breeding**		
4	Observation of a male and female (pair) during the breeding season	“nonbreeding”
5	Observation of a male and female during the breeding season on two or more days	“nonbreeding”
6	Courtship behavior or copulation	“likely breeding”
7	Individuals observed visiting a probable nest site	“likely breeding”
8	Behavior of individuals indicates nearby nest location	“likely breeding”
9	Only during ringing: brood patch visible	“likely breeding”
10	Nest building observed (at nest location)	“likely breeding”
**Confirmed breeding**		
11	Distraction behavior indicating nearby (but unobserved) nest or offspring	“likely breeding”
12	Currently occupied nest (includes inactive nests)	“likely breeding”
13	Fledglings that are yet incapable of sustained flight	“likely breeding”
14	Occupied nest, but contents not observed. Adults enter and remain inside the nest before leaving or exchanging duties	“likely breeding”
15	Adult carrying a fecal sac	“likely breeding”
16	Adult carrying food to provision offspring	“likely breeding”
17	Eggshell below nest	“likely breeding”
18	Nest with incubating adult	“likely breeding”
19	Nest with eggs or nestlings	“likely breeding”
**If other codes cannot be applied**		
30	Possible breeding	“nonbreeding”
40	Probable breeding	“likely breeding”
50	Confirmed breeding	“likely breeding”
99	No observation despite an active search for the species	Excluded from analysis

## Appendix 3

**TABLE A2:** Overview of the model structure for the start, end, and duration of the breeding season.

Start of the breeding season	Start ~ temperature (prebreeding) * precipitation days (prebreeding) + snowmelt timing + (1|year) + (1|biogeographic region)
End of the breeding season	End ~ temperature (during breeding) + snowmelt timing + snowmelt duration + (1|year) + (1|biogeographic region)
Duration of the breeding season	Duration ~ temperature (prebreeding) * precipitation days (prebreeding) + temperature (during breeding) + (1|year) + (1|biogeographic region)

*Note:* Asterisk defines an interaction effect, plus signs defines an additive effect, and random effects (random intercept) are specified in brackets (1|..).
